# The Willingness-to-Pay for General Practitioners in Contractual Service and Influencing Factors among Empty Nesters in Chongqing, China

**DOI:** 10.3390/ijerph120809330

**Published:** 2015-08-10

**Authors:** Fei Chen, Xiang-Long Xu, Zhan Yang, Hua-Wei Tan, Liang Zhang

**Affiliations:** 1 School of Medicine and Health Management, Tongji Medical College, Huazhong University of Science & Technology, Wuhan 430030, China; E-Mail: chenfei6639@126.com; 2 School of Public Health and Management, Chongqing Medical University, Chongqing 400016, China; E-Mails: xianglong1989@126.com (X.-L.X.); tanhuawei-2009@163.com (H.-W.T.); 3 College of Foreign Languages, Chongqing Medical University, Chongqing 400016, China; E-Mail: zenith_fat@126.com

**Keywords:** contractual service, willingness-to-pay, general practitioners, empty nesters, Contingent Valuation Method, Cox’s proportional hazards regression model

## Abstract

*Background:* In 2012, a pilot health policy of contractual service relations between general practitioners and patients was implemented in China. Due to the decline in body and cognitive function, as well as the lack of family care and narrow social support networks, the demand of health services among the elderly is much higher than that among the general population. This study aims to probe into the empty nesters’ willingness-to-pay for general practitioners using a contractual service policy, investigating empty nesters’ payment levels for the service, and analyze the main factors affecting the willingness of empty-nesters’ general practitioners using contractual service supply cost. *Methods:* This cross-sectional study adopted a multistage stratified sampling method to survey 865, city empty nesters (six communities in three districts of one city) aged 60–85 years. A condition value method was used to infer the distribution of the willingness-to-pay; Cox’s proportional hazards regression model was used to analyze the influencing factors of willingness-to-pay. *Results:* More than seventy percent (76.6%) of the empty nesters in this city were willing to pay general practitioners using contract service in Chongqing. The level of willingness-to-pay for the surveyed empty nesters was 34.1 yuan per year. The median value was 22.1 yuan per year, which was below the Chongqing urban and rural cooperative medical insurance individual funding level (60 yuan per year) in 2013. Cox’s proportional hazards regression model analysis showed that the higher the education level was, the worse the self-reported health status would be, accompanied by higher family per capita income, higher satisfaction of community health service, and higher willingness-to-pay empty nesters using a contract service. Women had a higher willingness-to-pay than men. *Conclusions:* The willingness-to-pay for general practitioners by contractual service is high among city empty nesters in Chongqing, thus, individual financing is feasible. However, people are willing to pay less than half of the current personal financing of cooperative medical insurance of urban and rural residents. Education level, family per capita income, and self-reported health status are the main factors affecting the cost sharing intention for general practitioners using contract service supply. According to the existing situation of different empty nesters, it is important to perfect the design of general practitioners using a contractual service policy system, according to differentiated personal financing levels.

## 1. Introduction

There is a rapidly aging population in the Asia-Pacific region, which increases the expenditures in public health. Especially in the reform of the medical insurance system for the elderly, cost control has become an urgent problem, which is difficult to solve. In order to be able to achieve the highest standards of physical and mental health, the ageing population must acquire affordable health care services suitable for their age, including prevention, treatment, and long-term care [1].

Empty nesters are a distinctive feature of the aging population in China, which account for 51.1% of the elderly in China [2]. The long distances and waiting times for medical services, and living without children greatly limit empty nesters’ access to health services in China. In the background of limited health resources and the rapid development of China’s ageing population, to ensure that the elderly get convenient and available medical and health services, optimize the elderly life quality, and improve elderly self care ability have become the focus in health for all countries [3]. Effective health management is an important approach to improve the elderly health level and control medical expenses [4,5]. Previous studies have shown that general practitioners, using contractual services, can effectively improve the health of urban community empty-nesters, promote quality of life and social support, and reduce loneliness and depression [6,7].

Contractual services, mainly based on general medical practitioners, rely on a general practitioner team, and take family as a vital unit. In the form of contractual services, general practitioners using contractual services provide a safe, effective, continuous, and basic medical and public health services to realize comprehensive health management [8–10]. General practitioners using a contractual service, mainly, play a role in reasonably utilizing health resources, reducing medical costs, and improving health conditions [11]. However, current, personal willingness-to-pay for general practitioners using a contract service piloted in China is not clear, and the individual level of willingness-to-pay is not clear either, with simple financing channels. Studies on financing mechanisms of Chinese general practitioners using a contract service are still in the theoretical stage, lacking empirical research [12,13]. 

In the context that general practitioners using the contract service community are extensively developing in China, Chongqing, as the area with the highest degree of ageing in China, is facing the problem of how to design a funding level of general practitioners using a contract service.

What are the main factors affecting the willingness-to-pay of general practitioners using a contract service? This study investigated city empty nesters in Chongqing, and used the Cox’s proportional hazards regression model to analyze the influencing factors of general practitioners using contractual service willingness-to-pay. General practitioners, under contract from the government to perfect community service provided the basis for personal financing mechanisms. This will help to promote the progress of general practitioners using contractual services in developing countries, improve the system of medical insurance policies, and make it meet the demand of elderly people.

## 2. Materials and Methods

### 2.1. Study Population and Design

Study participants were empty nester representatives. Data were collected through questionnaire survey from March to August in 2014, in the following three areas: Yuzhong District, Yubei District, and Dian Jiang District. 

### 2.2. Ethical Approval

All subjects gave their informed consent for inclusion before they participated in the study. The study was conducted in accordance with the Declaration of Helsinki, and the protocol was approved by the Ethics Committee of Chongqing Medical University (2014059). 

### 2.3. Questionnaire

A standardized questionnaire was designed based on factors which affected the willingness-to-pay of general practitioners by contractual service among empty nesters that covered general demographic characteristics (gender, age, education level, occupation, marital status), social and economic characteristics (family per capita income, self-reported family burden of diseases, social support), health condition (number of suffering from chronic diseases, self-reported health status), evaluation of general practitioners’ service (knowledge degree of general practitioners by contractual service, service selection desire of general practitioners using contractual service, and degree of satisfaction of community health service), and the methods of pension (home pension, Institution pension).

### 2.4. Data Collection

Data were collected through a cross-sectional survey from March to August in 2014. The participants were empty nesters, aged over 60, in communities of Chongqing. This city has the highest degree of ageing in China. Multi-stage random sampling method was used, the selected districts covering different economic development levels: Yuzhong District, Yubei District, and Dianjiang County. Then, we randomly selected two communities from each district and county, each community involving 150 empty nesters, and a total of 900 urban empty-nest elderly were surveyed. Through a structured questionnaire to collect data, the questionnaire included two parts: (1) Demographic characteristics, social and economic characteristics, and the situation of community health services, pension status, *etc*. (2) Willingness-to-pay of general practitioners using contractual services. To effectively reduce the understanding deviation, we conducted face-to-face interviews.

### 2.5. Data Analysis

The questionnaires data were carefully checked before being put into the database, using Epi-data3.1 software. After a meticulous sorting and data cleaning, analyses were performed using Spss19.0 software. Missing data were excluded and all data entries were double-checked in order to prevent errors. χ^2^ test was used to compare differences in categorical variables. All statistics were performed using a two-sided test and statistical significance was considered at *p* < 0.05. The distribution of the value method of willingness-to-pay was derived by using the COX proportional hazards model to analyze the influence factors of willingness-to-pay.

#### 2.5.1. Contingent Valuation Method (CVM)

Previous studies show that it was feasible to design general practitioners using contract service personal funding level according to medical insurance personal funding levels [14,15,16].

In 2013 and 2014, Chongqing medical insurance fund-raising standard was 300 yuan per year, the government covering 240 yuan per year, and insurance 60 yuan per year. When designing the questionnaire, in the personal pay for general practitioners using contract services, with reference to medical insurance funding level, the personal financing level was up to 60 yuan per year. In order to reduce the starting deviation of CVM, this research referred to the current level of medical treatment insurance in Chongqing, building virtual general practitioners using the contractual insurance market environment. The multiple boundary two-fraction selection method of one-way diminishing inquiry mode was adopted for general practitioners using contractual service willingness-to-pay related data. 

#### 2.5.2. Cox’s Proportional Hazards Regression Model

Cox’s proportional hazards regression model, was adopted to study the influencing factors of empty nesters using contract payment [17,18]. The Cox’s proportional hazards regression model is defined as:

(1) $$h_{i}(b,X_{i})=h_{0}(b)\exp\sum_{j=1}^{p}X_{ij}\beta_{j}(i=1,2,3,...,n;j=1,2,3,...,p)$$

where n is the number of samples, p is the number of introduced covariates, h0 (b) is the baseline hazard at economic burden b, representing the hazard for a person with the value 0 for all the predictors X. h_i_(b,X_i_) indicates the risk function of the i^th^ empty nesters in the economic burden level b exposed to the Xi, namely, the empty nesters has no intention to use contract payment when he/she is in the economic burden level b, and if less than level b, the risk function represents the conditional probability of willingness to pay; $\exp\sum_{j=1}^{p}X_{ij}\beta_{j}$ is the relative risk function; β_j_ expresses the impact of a one-unit change of predictors Xi on the nature logarithm of the relative risk that the empty nesters refuse to pay for the service.

β_j_ is negatively correlated to the willingness-to-pay, that is β_j_ greater than 0 shows that the corresponding predictor would decrease willingness-to-pay; β_j_ less than 0 shows that the corresponding predictor would increase willingness-to-pay. All the statistical analyses described above were carried out using SPSS21.0 statistical software. 

#### 2.5.3. Probability Unit Method

This study conducted probability analysis for the range of willingness-to-pay, the median value was calculate based on half-effect quantity, under the condition of the elderly people surveyed had pay willingness [19]. The model is as follows:

(2) Pr(v)=A+BLnWTP

where v is target variable of willingness-to-pay; Pr (v) is the probability that the elderly have a willingness to pay; A is the natural response rate that the elderly have to pay to; A and B are the model parameters; Covariate LnWTP's willingness to pay (WTP) is to be converted into the natural logarithmic form.

## 3. Results

### 3.1. General Description of the Sample

Among the 900 interviewees, the response rate was 865 (96%), including 371 (42.9%) men and 494 (57.1%) women. Interviewees were aged 60 to 85, and the average age was 69.6 years. The education level of 567 (65.6%) interviewees was primary schooling or below; 298 (34.4%) were of middle schooling or above. Retired occupational structure was farmers (40.1%), employees (29.9%), and institution staff (16.3%). Three hundred and fifteen (36.4%) of people’s family per capita monthly income was above 2000 yuan; 342 (39.5%) persons were between 1000–2000 yuan; 208 (24.1%) people were below 1000 yuan. Five hundred and ninety-six (68.9%) interviewees had at least one chronic disease, and 269 (31.1%) suffered from no chronic diseases.

### 3.2. The Willingness-to-Pay of General Practitioners Using Contractual Service

According to the results, the payment level of general practitioners using contractual services fell into seven half-open intervals, six complete intervals, respectively, were (0,10), (10,20), (20,30), (30,40), (40,50), (50,60) and a censored interval (60+ up). Results showed that 663 (76.6%) were willing to pay for general practitioners using contractual services. Payment levels falling in the full range of samples were 791 (91.6%). The willingness-to-pay of general practitioners using contractual services is shown in Table 1.

**Table 1:** The interval distribution of general practitioners by contractual service.

Payment Level	Method 1 ^2^		Method 2 ^3^	
	Number/Frequency	Cumulative Frequency/Frequency	Number/Frequency	Cumulative Frequency/Frequency
(0,10) ^1^	232/26.8	865/100	30/4.5	663/100
(10,20)	90/10.4	633/73.2	90/13.6	633/95.5
(20,30)	177/20.5	543/62.8	177/26.7	543/81.9
(30,40)	122/14.1	366/42.3	122/18.4	366/55.2
(40,50)	98/11.4	244/28.2	98/14.8	244/36.8
(50,60)	73/8.4	146/16.8	73/11.0	146/22.0
(60,+∞ )	73/8.4	73/8.4	73/11.0	73/11.0
Total	865/100	−	663/100	−

Notes: **^1^** If empty nesters are willing to pay but do not accept any given price level, we set that willingness-to-pay as 0; If empty nesters are not willing to pay, we set that willingness-to-pay as 0; **^2^** Method 1 (0, 10) range includes being willing to pay but at any given price level not being willing to accept the service and the total number of the elderly were not willing to pay; **^3^** Method 2 (0,10) range contains the number of elderly only being willing to pay but at any given price level not being willing to accept.

### 3.3. The Prediction of Pay Levels on General Practitioners by Contractual Service

We used probit analysis to calculate the median value of willingness-to-pay. Probit analysis model under two kinds of method of parameter estimation results were obtained using 20 iterations. According to the results of two methods probit analysis, models under the goodness-of-fit of the chi-square statistic were 90.89 and 87.34, respectively, which were at 1% (*p* < 0.01) significance at the same level of statistics. Probit analysis model parameter estimation results are shown in Table 2.

**Table 2:** The probability of payment interval unit analysis.

Parameter	Method 1				Method 2			
	Estimate	SE	z	95% CI	Estimate	SE	z	95% CI
B	−1.077 *	0.033	−32.735	(−1.142, −1.013)	−2.065 *	0.064	−32.441	(−2.190, −1.940)
A	3.332 *	0.112	29.754	(3.220,3.444)	7.289 *	0.230	31.697	(7.059,7.519)

Note: * *p* < 0.01.

Probit analysis model parameter estimation into (2) generates the general practitioners using contractual service pay levels, and each level under the confidence level of 95% confidence interval of the upper and lower limits (see Table 3). Results show that, with method 1, the calculated median value of urban community elderly general practitioners using contractual service payment levels was 22.052 yuan per year, and, in method 2, payment levels were 34.104 yuan per year.

**Table 3:** Payment level measurement among empty nesters.

Cumulative Probability	Method 1		Method 2	
	Estimated Value	95% CI	Estimated Value	95% CI
0.30	35.882	(28.392,49.523)	43.963	(41.886,46.389)
0.35	31.535	(24.778,41.687)	41.100	(39.239,43.197)
0.40	27.899	(21.492,35.869)	38.556	(36.843,40.416)
0.45	24.780	(18.491,31.412)	36.244	(34.624,37.939)
0.50	22.052	(15.770,27.874)	34.104	(32.534,35.690)
0.55	19.623	(13.330,24.958)	32.091	(30.536,33.611)
0.60	17.430	(11.160,22.460)	30.167	(28.604,31.654)
0.65	15.420	(9.239,20.248)	28.300	(26.711,29.778)
0.70	13.552	(7.540,18.226)	26.457	(24.831,27.944)

### 3.4. General Practitioners Using Contractual Service Influencing Factor Analysis of Willingness-to-Pay

Statistical descriptions of the variables are shown in Table 4.

**Table 4:** Variable definition and statistical description.

Variable Categories	Code	Variable Name	The Definition of the Variables and Measurements	Mean Value	Standard Deviation
Willingness-to-pay	y	Willing to pay	The general practitioners type of pension insurance’ willing to pay **^1^**	–	–
Demographic characteristics	X1	Gender	Male = 1, Female = 0	0.429	0.495
	X2	Age	Actual age	69.558	6.976
	X3	Education level	Primary schooling and below = 1, junior middle schooling = 2, High schooling and above = 3	1.501	0.751
	X4	Occupation before retire	Public institution personnel = 0, enterprise staff = 1, individual businessmen = 2, famer = 3	1.983	0.836
	X5	Marital status	Married=1, Other = 0 **^2^**	0.845	0.362
Physical condition	X6	The number of chronic diseases	Actual kind of chronic diseases	1.025	0.940
	X7	Health status by self-reported	Poor = 1, General = 2, Well = 3	2.186	0.781
Socioeconomic characteristics	X8	Family disease financial burden by self-reported	Cannot stand=1,Can stand in general = 2, Can stand = 3	2.123	0.769
	X9	Social support	one type support = 1, two types of supports = 2, three types of supports = 3 **^3^**	1.871	0.364
	X10	Family per capita income	Under 1000 yuan = 1, 1000–2000 yuan = 2, Above 2000 yuan = 3	1.810	0.621
Community doctor service	X11	Awareness of general practitioners by contractual service	Know = 1, Do not know = 0	0.813	0.389
	X12	The choice of general practitioners by contractual service	Do not choose = 1, Not sure = 2, Choose = 3	2.221	0.831
	X13	Community health service satisfaction	Not satisfied = 1, Indifferent = 2, Satisfied = 3	2.552	0.614
Pension mode	X14	The choice of old-age care	Traditional family caring =1 **^4^**, in institutions = 0	0.787	0.410

Notes: **^1^**^.^ This research adopts the contingent valuation method (CVM) the one-way decreasing inquiry mode for general practitioners service data of willingness-to-pay. If consumers’ willingness-to-pay are located in (+ up, 60), the willingness-to-pay = 7; if consumers’ willingness-to-pay are located in (0,10), the willingness to pay = 1, and so on; **^2^**^.^ Other includes four cases: single, divorced, separated, and widowed. **^3^**^.^ Social supports include social support, offspring support and other support, the actual kind being counted. **^4^**^.^ Home endowment refers to endowment place in the family pension, including the traditional family endowment and community endowment.

### 3.5. COX Proportional Hazards Regression Analysis

From the test of goodness of fit, the card party statistic model coefficient was 39.084; −2logarithmic likelihood value was 5243.276, *p* = 0.000; explaining model fitting effect was good. COX proportional hazards regression model showed that gender, level of education, self-reported health status, family per capita income, and community health service satisfaction affect general practitioners use of contractual service willingness-to-pay, the specific impact degree was as follows: The education level was positively correlated with the empty nesters’ willingness-to-pay for general practitioners in contract services; the self-evaluation in health was negatively correlated with the empty nesters’ willingness-to-pay for general practitioners in contract services; the family per capita income was in direct proportion to the empty nesters’ willingness-to-pay for general practitioners in contract services; the satisfaction of community health service was in direct proportion to the empty nesters’ willingness-to-pay; In addition, women had a higher willingness to pay than men, as shown in Table 5.

## 4. Discussion

This study finds that the willingness-to-pay of general practitioners using contractual services and by influencing factors among empty nesters is high. The implementation of general practitioners using contractual services in developing countries is feasible. However, people pay less than half of the individual financing of the cooperative medical insurance for urban and rural residents. Education level, family per capita income, and self-reported health status factors are the main factors of cost sharing intention, influencing general practitioners using contract service supply.

**Table 5:** COX proportional hazards model estimation.

Influence Factors	B	SE	Wald	Exp (B)	95% CI (Exp (B))	Significance Level (Sig.)
X1	0.106	0.04	7.152	1.112	(1.029,1.193)	0.025
X2	−0.004	0.005	0.620	0.996	(0.986,1.006)	0.246
X3	−0.162	0.056	8.429	1.176	(1.054,1.312)	0.014
X4	−0.126	0.072	3.066	0.881	(0.765,1.015)	0.262
X5	0.077	0.046	2.8	1.037	(0.960,1.121)	0.066
X6	0.037	0.040	0.864	0.926	(0.846,1.013)	0.329
X7	−0.206	0.098	4.431	1.228	(1.014,1.487)	0.003
X8	−0.003	0.051	0.003	0.997	(0.902,1.102)	0.441
X9	−0.159	0.095	2.789	0.853	(0.708,1.028)	0.072
X10	−0.265	0.061	18.806	0.768	(0.681,0.865)	0.000
X11	−0.140	0.089	3.508	0.869	(0.730,1.034)	0.068
X12	0.008	0.042	0.034	1.008	(0.928,1.094)	0.331
X13	−0.199	0.054	13.858	0.819	(0.738,0.930)	0.000
X14	0.078	0.088	0.788	1.082	(0.910,1.286)	0.153
Goodness of fit test	Chi-square = 39.084		*p* = 0.000	−2LogLikelihood = 5243.276		

By 2050, nearly 80% of the elderly people in the world will live in developing countries, and China is a typical example. Currently, except for a few countries, developing countries in the Asian-Pacific area have little private health insurance. Families are mainly responsible for taking care of, and financially supporting, their elderly relatives [20]. Related research focuses on integrating medical resources and social resources for elderly services, forming the government–market–volunteer tripartite coordination financing mechanisms; additionally, relevant research points out that general practitioners using contractual services are paid by the government, basic medical insurance, society, and the individual [2]. In developing countries, the development of general practitioners using contractual services has a certain model function. In China, general practitioners provide basic medical services for signing residents and a fee for health services were paid for according to the number of contractors by the year. The government service charge is shared by medical insurance funds, the basic public health services funds, and the signed, individual citizens. The specific criteria and the safeguard scope are decided by the local health service level, signing population structure, and funds for basic medical insurance and public health bearing ability [8]. However, currently, in the practice of Chinese general practitioners using contractual service, payment mechanisms are not unified in each province, and personal levels of willingness-to-pay are not clear.

Previous studies have shown that general practitioners using contractual services can effectively improve the health of urban, community empty-nesters, promote quality of life and social support, and reduce the level of loneliness and depression [7]. General practitioners using contractual services are a trend in the development of the community health service model, and the ultimate goal is to maintain healthy residents, improve public health service ability, and reduce medical costs. Carrying out general practitioners using contract services mainly lies in the corresponding safeguarding of funds [21]. The results show that more than seventy percent of urban empty-nesters are willing to pay general practitioners using a contract service supply cost; most visible empty-nesters have a willingness to pay for general practitioners’ services, showing that general practitioners using contract service personal financing is feasible.

The results show that empty nesters’ willingness-to-pay for general practitioners using contractual services has the following characteristics: females have a higher willingness-to-pay than males. The education level, family per capita income and satisfaction of community health service was positively correlated with willingness-to-pay of empty nesters for general practitioners using contractual service;the self-evaluation in health was negatively correlated with the empty nesters’ willingness-to-pay for general practitioners in contract services.

Women are more willing to pay more attention to their own health than men by the female’s special physiological and psychological characteristics, thus, their willingness-to-pay is higher than males [22]. Education level is an indicator reflecting social and economic status. The higher an education level is, the higher family economic income level will be, and, hence, a higher ability to pay and higher willingness-to-pay [23]. Self-reported health status is a subjective evaluation from various aspects. The poorer the status is, the greater the demand for health services will be [24], thus, there is a strong willing to pay. Differences in social and economic status change people with respect to health service utilization. A higher family per capita income means stronger payment ability, hence a higher willingness-to-pay [25,26]. Therefore, the financing system design should consider the different groups of empty nesters, including demographic characteristics, physical health, and social and economic characteristics, so as to provide differentiated services and set the differentiation of the funding levels.

Based on the actual situation of empty nesters, this study puts forward the suggestion that we should perfect the design of a policy system of general practitioners using contract services, and divide the differentiation of personal financing levels. This study has a strong practical significance to improve the supply efficiency quality, and elderly satisfaction of general practitioner in contract service, in developing countries. This would help to accelerate general practitioners using contract service schedules in developing countries, render the ageing population up to the highest standards of physical and mental health, and improve the system of medical insurance policies, so as to meet the needs of elderly people.

This study also has some limitations that must be addressed. First, cross-sectional survey data reduced the ability of the study to make direct causal inferences, explore whether or not unmeasured factors can better explain the observed relationships, and determine the direction of causality. Second, this study did not assess the concurrent validity of the questionnaire. This questionnaire is of our own design. We did not assess the validity of the questionnaire with measures, which may limit the validity of the findings. In future study, if providers are able to offer a variety of standardized products, which patients can prospectively buy, also needs to be asked.

## 5. Conclusions

The willingness-to-pay of general practitioners using contractual services is high among city empty nesters in Chongqing, thus individual financing is feasible. However, people pay less than half of the individual financing of cooperative medical insurance of urban and rural residents. Education level, family per capita income, and self-reported health status are the main factors affecting the cost sharing intention of general practitioners using contract service supply. According to the existing situation of different empty nesters, it is important to perfect the design of general practitioners using contractual service policy systems, according to differentiated personal financing levels. 
